# Phylogeny and herbivory are related to avian cecal size

**DOI:** 10.1038/s41598-019-40822-0

**Published:** 2019-03-12

**Authors:** Andrew Hunt, Layla Al-Nakkash, Andrew H. Lee, Heather F. Smith

**Affiliations:** 1 Department of Biomedical Sciences, Midwestern University, Glendale, AZ 85308 USA; 2 Department of Physiology, Midwestern University, Glendale, AZ 85308 USA; 3 Department of Anatomy, Midwestern University, Glendale, AZ 85308 USA; 4 School of Human Evolution and Social Change, Arizona State University, Tempe, AZ 85287 USA

## Abstract

Avian ceca, a pair of blind sacs arising from the junction of the ileum and colon, are homologous to the cecum in mammals. Cecal size is hypothesized to depend on dietary proclivities and pressures, with faunivorous species having short ceca, whereas herbivorous species have long ceca. Previous tests of this hypothesis, however, did not account for phylogenetic pseudoreplication among closely related taxa. We collated published data on cecal length, dietary category, flying ability, and body mass from 155 avian taxa. Character states were mapped onto a phylogenetic framework, and the permutation tail probability test was used to detect phylogenetic signal in each character. Phylogenetic signal is significant among the characters. As with the cecoappendicular complex in mammals, closely-related birds tend to have similar cecal length. To account for phylogenetic pseudoreplication, we performed phylogenetic generalized least squares regression on cecal length and body mass with dietary category, superordinal-level clade, and flying ability as cofactors. The best-fitting regression model supports the dietary hypothesis for the avian cecum. Among sampled birds of comparable body mass, mean cecal length is significantly longer in herbivorous species than in carnivorous ones (*p* = 0.008), presumably allowing the extraction of nutrients without the burden of fermenting bulky masses of dietary fiber. Exceptions to this trend, however, suggest that avian ceca are functionally complex and may have additional roles in water balance and nitrogen recycling.

## Introduction

The avian ceca are a pair of blind sacs that arise from the junction of the ileum and colon, and may extend alongside the ileum^1–3^. These structures are highly variable among species of birds. For example, the ceca are short in the Columbidae (pigeons and doves), but they can project a large distance along the ileum in the Galloanserae (fowl)^4^. However, the causes for this variation remain poorly understood. A homologous structure is found in many mammalian species, the cecum. Smith and colleagues recently proposed the term “cecoappendicular complex” to reflect the fact that the mammalian cecum evolves in concert with its attached appendix^5^. The cecoappendicular complex has been recently shown not to correlate with diet, social behavior, or any other ecological factor across mammals^5,6^. Instead, the mammalian cecoappendicular complex exhibits significant phylogenetic signal, such that new phenotypes appear restricted primarily by phylogenetic conservatism across mammals (i.e., the tendency of closely-related species to resemble one another irrespective of the adaptive landscape)^5–7^. This intriguing finding raises the question as to whether the homologues in birds, the ceca, share similar evolutionary conservatism.

Avian ceca span a wide range of morphological types, from essentially absent to small and lymphoid to large and glandular^1,4^. They serve various diverse functions, including fermentation, water absorption, digestion, and immunity, which may be performed to varying degrees in different species and cecal types. In herbivorous birds that feed on fiber-rich plant material, ceca house microorganisms that presumably assist in the breakdown of fiber through fermentation via anaerobic degradation to ammonia and volatile fatty acids^8,9^. In order for nutrient absorption to take place in the ceca, particles are refluxed from the rectum into the ceca along with digestive fluid and urine^3,10,11^. Retrograde urine transport therefore directs uric acid to the site of fermentation, and uric acid is likely to be a major substrate for fermentation. Most microorganisms found within the avian ceca are able to degrade urea and uric acid into carbon dioxide and ammonia, which can be rapidly absorbed and utilized for the production of amino acids and protein^3,12–14^. Maintenance of water balance is another vital function that may be performed by the avian ceca, because sodium and water are reabsorbed there in great abundance^15–17^. Many species also possess a cecal tonsil composed of lymphoid tissue, suggesting the ceca play a role in immune defense^18^. B and T cells, avian immunoglobulins (IgA, IgG, and IgM), and germinal tissue have been found in the cecal tonsil^19–21^.

In two studies^1,4^ large surveys of the occurrence of cecal types were conducted across birds. These studies grouped birds by taxonomic order and compiled a list of the occurrence of cecum found in each group, noting the appearance, size, shape, and histological type for each order. Clench & Mathias conducted a broad survey of the occurrence of cecal types, and found no strong correlation between cecal size and taxonomic order, except in those species that were closely related^1^. Another study by DeGolier and colleagues reviewed the cecal morphology of 154 species of birds and concluded that it is fairly consistent within some orders (e.g., Gruiformes, Cuculiformes, Strigiformes)^4^. However, both studies found that variation exists within some taxonomic orders, and that overall, diet most likely had the largest effect on cecal variation.

Yet, diet alone does not fully explain cecal morphology because some birds with similar diets have grossly different ceca^1^. For example, hawks and owls, which have very similar carnivorous diets, differ in cecal morphology. The ceca of owls are quite large and are of a glandular type, whereas those of hawks are smaller and lymphoid^1,8^. Clench and Mathias^1^, as well as DeGolier and colleagues^4^, noted that in some species, other factors (e.g., foodstuff digestibility, water availability, etc.) may be more influential on the presence of ceca than diet alone. Moreover, flying ability may affect cecal morphology; selection for strong flying ability may impose weight-saving constraints expressed in part by reducing cecal size^3,9^.

None of the preceding studies formally accounted for the effect of phylogeny. The study by DeGolier^4^, for example, grouped birds by taxonomy rather than by hierarchically-nested clades. Ignoring phylogeny in this way can potentially obfuscate the relationship between form and function^22^. In fact, a study on models that incorporated phylogenetic information fit the data better than the nonphylogenetic ordinary least squares model^23^. It has been shown that the mammalian appendix has evolved statistically more times than would be expected by chance alone, suggesting that the appendix has some function in mammals^5–7^. By mapping morphological variation in the cecoappendicular complex onto a phylogenetic tree, Smith and colleagues^5,6^ identified where the appendix appeared and disappeared throughout evolutionary history. These studies illustrate how phylogeny can be incorporated into analyses of correlated character evolution. However, such an analysis has not yet been conducted on avian ceca to assess whether they depend on phylogeny, dietary category, or both.

Here, we exploit recent advances in comparative methods^22,24,25^ and avian systematics, such as improved species-level phylogenies based largely on molecular data^26–28^. These advances allow us to re-assess previous conclusions that avian cecal length depends mainly on dietary category^1,4^ and potentially on flying ability^3,9^ and phylogeny^4^.

## Results

### Assessment of phylogenetic signal

**Figure 1:**
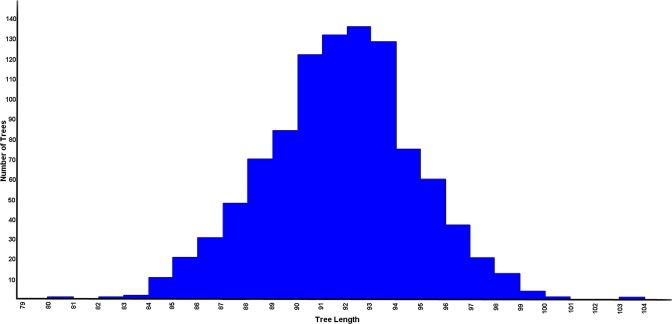
Plot of the distribution of 9999 randomly permuted trees showing the number of minimum evolutionary steps (tree length) in dietary category. None of the randomly permuted trees were found to have a tree length equal to or less than the original tree (44 steps) suggesting a highly significant phylogenetic signal in dietary category (*p* < 0.001).

In this sample of 155 avian taxa (Table S1), values of Pagel’s lambda for log-transformed body mass and log-transformed cecal length approximate one (*p* < 0.001), consistent with a close fit to Brownian motion evolution (Table 1). In addition, the permutation tail probability (PTP) tests on categorical variables reveal that none of randomly permuted trees had fewer steps than 46 for dietary category and 13 for flying ability (Fig. 1). Therefore, these tests confirm that each continuous and categorical character shows significant phylogenetic signal (*p* < 0.01).

**Table 1:** Phylogenetic signal in sampled categorical and continuous characters.

Character	λ	*p*-value
**Continuous characters**		
Body mass (ultrametric)	0.990409	6.30E-14
Body mass (unity-branch)	0.999934	5.30E-08
Cecal length (ultrametric)	1.035615	1.10E-54
Cecal length (unity-branch)	0.999934	5.90E-45
**Categorical characters**		
Dietary category	—	0.001

All characters were found to have a highly significant phylogenetic signal. Continuous characters were assessed using Pagel’s Lambda (**λ)**. Categorical characters were evaluated using permutation tail probability (PTP) tests.

### Phylogenetic comparative methods

Regression analysis reveals that models with phylogenetic weighting have substantially stronger evidential support than those without weighting (i.e., OLS: ordinary least squares). The AIC values of simple, additive, and interaction (multiplicative) models with phylogenetic weighting are substantially smaller (better supported) than corresponding OLS models (Table 2). This result is broadly consistent with assessments using Pagel’s lambda and the PTP test, and suggests that the data (specifically their residual values) have significant phylogenetic signal.

**Table 2:** Model comparisons of the effect of cofactors (clade, diet, and flight) on the relationship between log-transformed cecal length and body mass.

Allometric model	AIC	ΔAIC
**Simple allometry**		
**Model**		
OLS	349.7	165.8
Brownian	203.1	19.2
OU (α = 0.0583)	198.5	14.7
**Same slope, different intercepts (clade)**		
**Model**		
OLS	249.9	66.1
Brownian	211.5	27.6
OU (α = 0.155)	189.2	5.4
**Same slope, different intercepts (diet)**		
**Model**		
OLS	320.2	136.3
Brownian	199.8	16.0
OU (α = 0.063)	194.6	10.8
**Same slope, different intercepts (flight)**		
**Model**		
OLS	334.7	150.9
Brownian	206.6	22.7
OU (α = 0.060)	201.6	17.8
**Same slope, different intercepts (clade & diet)**		
**Model**		
OLS	236.7	52.8
Brownian	208.0	24.2
OU (α = 0.1697)	183.8	0*
**Same slope, different intercepts (clade, diet & flight)**		
**Model**		
OLS	237.6	53.8
Brownian	211.7	27.9
OU (α = 0.172)	187.1	3.3
**Different slopes and intercepts (clade)**		
**Model**		
OLS	256.6	72.8
Brownian	214.3	30.5
OU (α = 0.141)	197.5	13.6
**Different slopes and intercepts (diet)**		
**Model**		
OLS	320.0	136.1
Brownian	196.1	12.3
OU (α = 0.051)	194.2	10.3
**Different slopes and intercepts (flight)**		
**Model**		
OLS	338.0	154.2
Brownian	209.0	25.2
OU (α = 0.0587)	204.7	20.8

The best supported model (*) has the lowest Akaike Information Criterion (AIC) value and ΔAIC less than 3.

**Figure 2:**
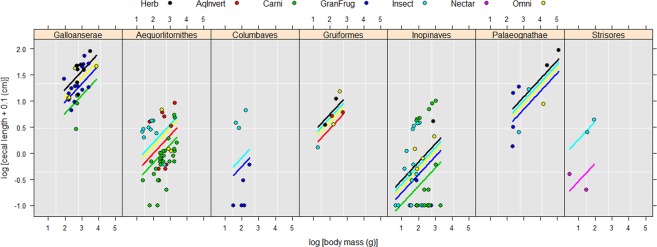
Cecal length depends on body mass, clade, and dietary factor based on phylogenetic generalized least squares regression. Abbreviations for dietary categories are as follows: AqInvert = Carnivore- Aquatic invertebrates; Carni = Carnivore; GranFrug = Granivore/Frugivore; Herb = Herbivore; Insect = Insectivore; Nectar = Nectarivore; Omni = Omnivore.

Based on ΔAIC values (Table 2), the best-supported model of log-transformed cecal length on log-transformed body mass has Ornstein-Uhlenbeck-weighting (α = 0.1697) with superordinal-level clade and dietary category as additive cofactors (Fig. 2). Each clade and dietary category has the same slope (0.348 ± 0.074). It is not significantly different from 0.33 (*df* = 141; *p* = 0.81), consistent with isometric scaling of linear dimensions on body mass. In addition to body mass, there is a phylogenetic effect on cecal length (Table 3). Galloanserae has significantly longer ceca than Aequorlitornithes (*p* < 0.0001), Columbaves (*p* = 0.0008), or Inopinaves (*p* < 0.0001). Conversely, Inopinaves has significantly shorter ceca than Gruiformes (*p* = 0.005), Aequorlitornithes (*p* = 0.02), or Palaeognathae (*p* = 0.009). Within each clade, some dietary categories further influence cecal length (Table 3). Specifically, ceca are significantly longer in herbivorous, insectivorous, and omnivorous birds than in carnivorous ones (*p* = 0.008, 0.0045, 0.0284, respectively).

**Table 3:** Regression coefficients for the best model between log-transformed cecal length and body mass with clade and dietary category as additive cofactors (*N* = 155).

Coefficient	Value	SE	*p*-value
**Reference levels: Inopinaves & Carnivore – Vertebrates**			
Reference Intercept	−1.3243458	0.2437551	0
Herbivory	0.4588238	0.1714065	0.0083
Carnivore – Aquatic Invertebrates	0.1816623	0.1607742	0.2604
Granivore/Frugivore	0.2200885	0.1525698	0.1514
Insectivore	0.3865855	0.1339115	0.0045
Nectarivore	−0.4127116	0.4367179	0.3463
Omnivore	0.3192183	0.1441751	0.0284
Galloanserae	1.3928131	0.2156552	0
Aequorlitornithes	0.4458934	0.1930361	0.0223
Columbaves	0.1564579	0.3556094	0.6606
Gruiformes	0.9224868	0.3213177	0.0047
Palaeognathae	0.900098	0.3386575	0.0088
Strisores	0.8523487	0.4736716	0.0741
log_10_(body mass)	0.347821	0.0744129	0
**Reference levels: Galloanserae & Herbivore**			
Reference Intercept	0.5272911	0.2764784	0.0585
Carnivore – Aquatic Invertebrates	−0.2771615	0.2095927	0.1882
Carnivore – Vertebrates	−0.4588238	0.1714065	0.0083
Granivore/Frugivore	−0.2387353	0.1439841	0.0995
Insectivore	−0.0722383	0.1712176	0.6737
Nectarivore	−0.8715354	0.4495318	0.0545
Omnivore	−0.1396055	0.1611331	0.3877
Aequorlitornithes	−0.9469197	0.2196839	0
Columbaves	−1.2363552	0.3598717	0.0008
Gruiformes	−0.4703264	0.33038	0.1568
Inopinaves	−1.3928131	0.2156552	0
Palaeognathae	−0.4927151	0.3230623	0.1295
Strisores	−0.5404644	0.485988	0.268
log_10_(body mass)	0.347821	0.0744129	0

Default output of the regression analysis contrasts intercept values for clade and dietary categories with the reference intercept.

An alternative model with Ornstein-Uhlenbeck-weighting (α = 0.172) and flying ability as an additional additive cofactor has substantially less support than the best model (Table 2: ΔAIC = 3.3). At least in the current sample of birds, flying ability does not significantly affect cecal length (*p* > 0.051).

We could not assess a multiplicative model with different slopes for clade, dietary category, and flying ability due to the small sample size of some categories. However, multiplicative models, each with a single cofactor (either clade, dietary category, or flying ability) generally have less support than corresponding additive models with a single cofactor (Table 2).

## Discussion

**Figure 3:**
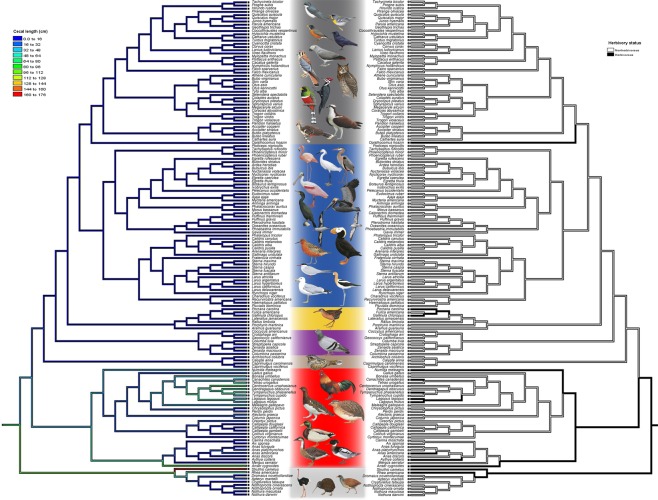
Mirror phylogenetic tree of 146 avian species suggesting poor correspondence between continuous cecal size (left) and herbivorous dietary group (right). PGLS analysis suggests that when accounting for differences in body size, cecal length is significantly longer in herbivorous species than in carnivorous ones (*p* = 0.003). Following Prum *et al*.^14^, the major neoavian clades are indicated in different colors: Aequorlitornithes (blue), Columbaves (purple), Galloanserae (red), Gruiformes (yellow), Inopinaves (grey), and Strisores (brown). Illustrations of representative bird species reproduced with permission from: del Hoyo, J., Elliott, A., Sargatal, J., Christie, D. A. & de Juana, E. (eds.) (2018). Handbook of the Birds of the World Alive. Lynx Edicions, Barcelona. (retrieved from http://www.hbw.com/ on May 11, 2018). This figure is not covered by the CC BY license. Credit to del Hoyo *et al*. (2018). All rights reserved, used with permission.

Cecal length, body mass, dietary categories, and flying ability each show phylogenetic signal. The presence of phylogenetic signal in these characters recommends caution when interpreting previous analyses of these characters based on traditional non-phylogenetic techniques. That stated, our results support the hypothesis linking long ceca to herbivory^4^. Phylogenetic regression demonstrates that when accounting for body size and clade, herbivory and relatively long ceca are correlated. As suggested in Fig. 3, herbivory evolved independently in Galloanserae and Palaeognathae; in both clades, herbivorous species tend to have long ceca (color-coded green to red). However, exceptions to the general trend (e.g., *Opisthocomus hoazin* and *Fulica americana*, and *Meleagris gallopavo*) suggest that for some species, additional factors other than dietary category may influence cecal length. For example, experimental studies in quail and grouse have shown that ceca elongate as a response to changes in food consumption rates rather than in fiber content^29,30^. The ceca filter large volumes of food, selecting the fibrous indigestible fraction for frequent excretion while retaining the nutrient-rich liquid fraction for additional processing and absorption. In this way, ceca may be an avian adaptation for efficient processing of ingested food^29^.

Alternatively as suggested by DeGolier and colleagues^4^, avian ceca may correlate with water balance and nitrogen recycling. To our knowledge, no phylogenetically-informed analyses have tested the water-balance and nitrogen-cycling hypotheses. Whereas herbivorous species are predicted have large ceca to filter and absorb the nutrient-rich fraction from bulky indigestibles, carnivorous species may also benefit from these organs, which may further process uric acid that forms as a waste product of high protein consumption. Thus, there may be several adaptive pressures selecting for large ceca and herbivory may simply be just one of them.

Interestingly, avian ceca show similar functional and evolutionary patterning to the mammalian cecoappendicular complex. Smith and colleagues^5,6^ tracked cecoappendicular evolution across mammals, and found no correlation between dietary category and any of the variables associated with the cecum or appendix, including appendix size, appendix presence, cecal morphology, or cecal size. Therefore, they concluded that dietary proclivities alone are not driving cecoappendicular evolution in mammals^5,6^, just as we have shown that diet alone is not driving cecal evolution in birds. Instead, both the mammalian cecoappendicular complex and avian colic ceca demonstrate significant phylogenetic signal, indicating that behavioral or body size characters are not independent of ancestry. Factors other than diet affect cecoappendicular size and shape, and this is likely true for birds as well. For example, accommodation also plays a role in determining appendix morphology, such that the appendix can change in size and histological composition throughout an individual’s lifetime. In humans, for example, the appendix reduces size and changes shape with age, due to loss of lymphoid tissue^31–33^. Future studies could investigate how heritable cecal accommodation is in birds to determine whether its role in the evolution of avian cecal morphology.

Previous studies have hypothesized that the constraints of flight may have led to reduced cecal size and fermentation capabilities in flighted birds^3,34^. Our analyses did not detect a correlation between cecal length and flying ability across the sample, suggesting that flight is not an inherently limiting factor for cecal length. It is possible, however, that other measures of cecal size and capabilities not included here, such as cecal volume, may be the variable limiting flight.

## Methods

### Sampling

We used the framework of a recently published avian phylogeny, which is based on conserved regions in 259 nuclear genes across 198 avian species^28^. Dense taxonomic sampling of non-passerine birds clarifies controversial relationships, particularly deep nodes towards the base of crown-group Aves^27,28^. For superficial nodes, we relied on the topology of a recent supertree containing approximately 5000 species^26^. This supertree was assembled using source trees that were recovered mostly from molecular data, with cytochrome b sequencing being the largest contributor (38.9% of source trees). Far fewer source trees came from morphological characters, with 0.004% of source trees based on digestive morphological data. A shortcoming of the supertrees is the lack of accurate branch-length information. In lieu of this information, we estimated branch lengths in our composite phylogenetic topology using Pagel’s method^35^, which generates arbitrary ultrametric (time-proportional) branches. Assembly of the composite phylogeny was performed using Mesquite Phylogenetic Software^36^ (v. 3.51).

Onto these phylogenies, we mapped morphological, dietary, and flight ability data from 155 avian species^4,37–46^ (Table S1). Morphological data consist of cecal length and body mass, both of which have strong right-skewed distributions that violate normality assumed by many statistical analyses. To better approximate the normal distribution, body mass and cecal length data were log-transformed. A small constant (0.1 cm) was added to cecal length data before log-transformation to avoid calculating undefined values. Primary dietary category was assigned for each species using cadaveric dissection and analysis of stomach contents (e.g., herbivore—greens, including the leaves of aquatic and terrestrial plants, comprise at least 50% of the stomach contents)^4^. In the absence of stomach contents, data from the literature were used. Primary dietary category was coded as a multi-state factor (herbivore, carnivore: aquatic invertebrates, carnivore: vertebrates, granivore/frugivore, insectivore, nectarivore, and omnivore). Flight ability was coded as a multi-state factor (strong flyer, weak flyer, flightless). All data collected and analyzed during this study are included in this published article (and accompanying Supplementary Information files, Data S1 and Table S1).

### Assessment of phylogenetic signal

Comparative data as described above are results of evolution and phylogenetic processes. Such data may show strong phylogenetic signal and violate the assumption that data points are statistically independent as required in many traditional statistical techniques (e.g., pairwise comparisons or regression)^22^. There are different methods to test for phylogenetic signal in continuous and categorical characters.

We assessed phylogenetic signal in continuous characters such as log-cecal length and log-body mass using the package “phytools”^47^ written for R^48^. This package implements Pagel’s lambda, which estimates the transformation needed to fit a Brownian phylogenetic model to the character data^49^. Pagel’s lambda typically ranges between zero (characters are independent of phylogeny) and one (characters evolved following Brownian motion); lambda greater than one is possible if traits are more similar than expected by Brownian motion. Unlike alternative measures of phylogenetic signal (e.g., Blomberg’s K), Pagel’s lambda seems robust to phylogenies with suboptimal branch-length information^50^ as is the case with the composite avian phylogeny. The null hypothesis (no phylogenetic signal) was rejected if the *p*-value was less 0.05.

To assess phylogenetic signal in categorical characters (diet and flight), we performed a permutation tail probability (PTP) test^51^ using Mesquite^36^. The minimum number of evolutionary changes (steps or tree length) required to explain the distribution of each character across the original tree was recorded. Two sets of randomized trees were generated using the settings uniform (yule) speciation (with a tree depth of ten) and parsimony character steps. The first set included 999 permutations and the second included 9,999 permutations. Character data were mapped onto the random topologies in each simulation and the number of evolutionary steps was recorded. This procedure permits the character distributions across the real phylogenetic tree to be compared to a random distribution, in order to determine whether the actual character distribution was significantly different than would be expected by chance alone. To calculate a *p*-value for each character, we counted the number of permutations with a tree length less than or equal to the observed length and divided by the total number of permutations^52^. Note that if none of the permutated data sets were as short or shorter than the observed values, we reported the *p*-value as 10 divided by the number of permutations—that is the probability of a Type I error is very small but not zero^53^. The null hypothesis (no phylogenetic signal) was rejected if the proportion of permutated data sets with tree lengths as short or shorter than the original data set was less than 0.05^54^.

### Phylogenetic comparative methods

We assessed relationships among cecal length, body mass, and various factors, using increasingly complex sets of regression models in the following order. In the first set, we evaluated log-transformed cecal length (dependent variable) regressed on log-transformed body mass (independent variable)—simple allometry. The second set consists of regressions in which categorical factors (i.e., dietary category, superordinal-level clade, and flying ability) were modeled as additive effects in isolation as well as in combination. The final set consists of models with the categorical factors as interactive (multiplicative) effects. Unfortunately, small sample size in various categories precluded the evaluation interactive effects beyond isolated factors.

Regressions were performed using phylogenetic generalized least squares (PGLS)^24,25^ with the “ape”^55^ and “nlme”^56^ package in R^48^. PGLS uses weighting to “correct” for non-independence of residual variation among data points^57^. Weighting is in the form of a variance-covariance matrix based on the sample phylogeny and model of evolution. Three models of evolution are commonly assumed in comparative analyses: (1) no evolution—all taxa are statistically independent^23^; (2) Brownian motion evolution—variance among related taxa increases linearly with time^58^; and (3) Ornstein-Uhlenbeck evolution—variance among related taxa increases exponentially with time^59^. We repeated PGLS regressions using each method of weighting.

Regression models differ in their complexity and fit to the data, so we compared them using Akaike’s Information Criterion (AIC). In general, the best supported model has the lowest AIC value^60^. Relative support between the best model and alternative models was assessed with difference (ΔAIC) values. Alternative models with ΔAIC values of at least 3, which is equivalent to a *p*-value of 0.051^61^, were rejected as substantially weaker than the best model.

## Supplementary information

Data S1

Table S1
