# Supplementary material for: Phylogeny and herbivory are related to avian cecal size
Source: Sci Rep. 2019 Mar 12;9:4243. doi: 10.1038/s41598-019-40822-0 (PMC6414633; doi:10.1038/s41598-019-40822-0)
Supplement: Supplementary file 2 — Table S1 [file 41598_2019_40822_MOESM2_ESM.pdf]

TITLE: Phylogeny and herbivory are related to avian cecal size

Andrew Hunt[1], Layla Al-Nakkash[2], Andrew H. Lee[3†], Heather F. Smith[3, 4†]*

[1]Department of Biomedical Sciences, Midwestern University, Glendale, AZ 85308.
[2]Department of Physiology, Midwestern University, Glendale, AZ 85308.
[3]Department of Anatomy, Midwestern University, Glendale, AZ 85308.
[4]School of Human Evolution and Social Change, Arizona State University, Tempe, AZ 85287.

*To whom correspondence should be addressed: Dr. Heather F. Smith, Department of Anatomy, Arizona College of Osteopathic Medicine, Midwestern University, 19555 N. 59th Ave., Glendale, AZ 85308, USA.
E-mail: hsmith@midwestern.edu, Tel: 1-623-572-3726, Fax: 1-623-572-3679.
[†]These authors contributed equally to this work.

Supplementary Information

Table S1: Faunivory status (Fauniv= faunivorous, Non-Fauniv= non-faunivorous), dietary category (I= insectivorous, Fr= frugivorous, O= omnivorous, Gr/Fr= granivorous/ frugivorous, C= carnivorous, H= herbivorous, N= nectarivorous, AI= aquatic invertebrates) relative and dichotomous cecal size, absolute cecal length, absolute intestinal length, body mass, and flight ability status of 146 taxa of birds. These data were then mapped onto a consensus phylogenetic supertree (Davis and Page, 2014). Data primarily from DeGolier and colleagues (1999) and web databases (Axelson et al., 2017; Myers et al., 2017).

Avian Taxon	Faunivory Status	Dietary Category	Relative Cecal Size (%)	Cecal Length (cm)	Intestinal length (cm)	Dichotomous Cecal Size	Body Mass (g)	Body Mass (g)	Flight Status
Tachycineta bicolor	Fa	I	Medium (2.5)	0.4	15.8	Small/Absent	Medium (15.4)	15.4	Flighted
Progne subis	Fa	I	Absent (0.0)	0	17.8	Small/Absent	Medium (30.3)	30.3	Flighted
Hirundo rustica	Fa	I	Medium (2.1)	0.3	14.6	Small/Absent	Medium (31.4)	31.4	Flighted
Piranga olivacea	Fa	I	Absent (0.0)	0	20	Small/Absent	Medium (34.8)	34.8	Flighted
Quiscalus quiscula	Non-Fa	O	Medium (1.2)	0.4	32.2	Small/Absent	Medium (81.1)	81.1	Flighted
Quiscalus major	Non-Fa	O	Medium (1.4)	0.7	49.2	Small/Absent	Large (178.2)	178.2	Flighted
Junco hyemalis	Non-Fa	Gr/Fr	Medium (1.2)	0.3	24.7	Small/Absent	Medium (28.6)	28.6	Flighted
Parula americana	Fa	I	Absent (0.0)	0	9.9	Small/Absent	Small (4.3)	4.3	Flighted
Geothlypis trichas	Fa	I	Absent (0.0)	0	11	Small/Absent	Small (6.3)	6.3	Flighted
Coccothraustes vespertinus	Non-Fa	Gr/Fr	Small (0.4)	0.2	51	Small/Absent	Medium (80.0)	80	Flighted
Hylocichla mustelina	Fa	I	Medium (2.0)	0.5	24.6	Small/Absent	Medium (51.7)	51.7	Flighted
Catharus ustulatus	Fa	I	Small (1.0)	0.2	20.2	Small/Absent	Medium (42.1)	42.1	Flighted
Turdus migratorius	Fa	I	Medium (1.1)	0.3	28	Small/Absent	Medium (76.6)	76.6	Flighted
Cyanocitta cristata	Non-Fa	O	Medium (3.0)	1.1	36.8	Small/Absent	Medium (61.0)	61	Flighted
Corvus corax	Non-Fa	O	Medium (1.8)	2	112.3	Small/Absent	Large (874.0)	874	Flighted
Lanius ludovicianus	Fa	I	Medium (1.1)	0.3	28.7	Small/Absent	Medium (28.7)	28.7	Flighted
Vireo flavifrons	Fa	I	Medium (6.7)	1	15	Small/Absent	Medium (20.0)	20	Flighted
Myiopsitta monachus	Non-Fa	Gr/Fr	Absent (0.0)	0	52.6	Small/Absent	Large (376.6)	376.6	Flighted
Psittacus erithacus	Non-Fa	Gr/Fr	Absent (0.0)	0	114	Small/Absent	Large (429.8)	429.8	Flighted
Cacatua galerita	Non-Fa	Gr/Fr	Absent (0.0)	0	130.5	Small/Absent	Large (589.0)	589	Flighted
Nymphicus hollandicus	Non-Fa	Gr/Fr	Absent (0.0)	0	41.4	Small/Absent	Medium (76.0)	76	Flighted

Selenidera spectabilis	Fa	I	Absent (0.0)	0	20.6	Small/Absent	Large (149.3)	149.3	Flighted
Colaptes auratus	Fa	I	Absent (0.0)	0	29.5	Small/Absent	Medium (78.9)	78.9	Flighted
Dryocopus pileatus	Fa	I	Absent (0.0)	0	51	Small/Absent	Large (178.3)	178.3	Flighted
Sphyrapicus varius	Fa	I	Absent (0.0)	0	28.7	Small/Absent	Medium (38.9)	38.9	Flighted
Megaceryle alcyon	Fa	C	Absent (0.0)	0	97.2	Small/Absent	Large (22.1.8)	22.1.8	Flighted
Coracias abyssinica	Fa	I	Large (15.0)	3.7	24.7	Large	Large (119.8)	119.8	Flighted
Trogon collaris	Fa	I	Medium (4.4)	3.2	72.7	Small/Absent	Medium (55.5)	55.5	Flighted
Trogon viridis	Fa	I	Large (15.5)	3.6	23.3	Large	Medium (90.5)	90.5	Flighted
Trogon violaceus	Fa	I	Medium (6.2)	2.8	45	Small/Absent	Medium (47.5)	47.5	Flighted
Coccyzus americanus	Fa	I	Large (12.0)	3.7	30.9	Large	Medium (46.0)	46	Flighted
Crotophaga ani	Fa	I	Medium (9.8)	2.9	29.7	Large	Medium (70.0)	70	Flighted
Geococcyx californianus	Fa	I	Large (14.2)	6.5	45.8	Large	Large (171.1)	171.1	Limited Flight
Archilochus colubris	Non-Fa	N	Medium (1.8)	0.1	5.6	Small/Absent	Medium (28.6)	28.6	Flighted
Calypte anna	Non-Fa	N	Medium (5.0)	0.3	6	Small/Absent	Small (3.0)	3	Flighted
Caprimulgus carolinensis	Fa	I	Large (15.6)	4.3	27.6	Large	Medium (89.1)	89.1	Flighted
Caprimulgus vociferus	Fa	I	Large (13.4)	2.4	17.9	Large	Medium (34.3)	34.3	Flighted
Pandion haliaetus	Fa	C	Small (0.3)	0.5	204	Small/Absent	XL (1104.5)	1104.5	Flighted
Accipiter cooperii	Fa	C	Small (0.2)	0.1	46.5	Small/Absent	Large (411.6)	411.6	Flighted
Accipiter striatus	Fa	C	Absent (0.0)	0	36	Small/Absent	Medium (81.6)	81.6	Flighted
Buteo platypterus	Fa	C	Small (0.2)	0.1	60	Small/Absent	Large (227.2)	227.2	Flighted
Buteo lineatus	Fa	C	Absent (0.0)	0	96.9	Small/Absent	Large (459.7)	459.7	Flighted
Falco sparverius	Fa	I	Small (0.1)	0	35.6	Small/Absent	Medium (87.7)	87.7	Flighted
Falco mexicanus	Fa	C	Absent (0.0)	0	65.5	Small/Absent	Large (338.0)	338	Flighted
Athene cunicularia	Fa	C	Large (13.4)	4.6	34.3	Large	Large (117.9)	117.9	Flighted
Bubo virginianus	Fa	C	Large (11.2)	9.9	88.4	Large	XL (1057.2)	1057.2	Flighted
Strix varia	Fa	C	Large (11.7)	8.9	76.2	Large	Large (651.0)	651	Flighted
Otus asio	Fa	C	Large (15.9)	4.4	27.9	Large	Medium (83.2)	83.2	Flighted
Otus kennicottii	Fa	C	Large (15.7)	4.1	26.2	Large	Medium (79.7)	79.7	Flighted

Tyto alba	Fa	C	Large (12.1)	6.9	57	Large	Large (419.6)	419.6	Flighted
Cathartes aura	Fa	C	Absent (0.0)	0	130.5	Small/Absent	XL (2097.0)	2097	Flighted
Podiceps nigricollis	Fa	AI	Medium (4.8)	6	125.4	Small/Absent	Large (347.4)	347.4	Flighted
Tachybaptus ruficollis	Fa	AI	Small (0.5)	0.4	79.5	Small/Absent	Large (202.2)	202.2	Flighted
Phoenicopterus minor	Fa	AI	Small (1.0)	3.2	317.8	Small/Absent	XL (1158.0)	1158	Flighted
Phoenicopterus ruber	Fa	AI	Medium (3.4)	9	266.5	Small/Absent	XL (1878.3)	1878.3	Flighted
Egretta rufescens	Fa	C	Small (0.9)	1.8	198	Small/Absent	Large (786.0)	786	Flighted
Butorides striatus	Fa	C	Absent (0.0)	0	91	Small/Absent	Large (178.8)	178.8	Flighted
Ardea herodias	Fa	C	Small (0.5)	1	193.1	Small/Absent	XL (1914.0)	1914	Flighted
Bubulcus ibis	Fa	C	Small (0.4)	0.2	55.2	Small/Absent	Large (220.7)	220.7	Flighted
Nyctanassa violacea	Fa	AI	Small (0.3)	0.4	137.1	Small/Absent	Large (511.0)	511	Flighted
Nycticorax nycticorax	Fa	C	Small (0.8)	1	127.9	Small/Absent	Large (486.9)	486.9	Flighted
Egretta caerulea	Fa	C	Small (0.6)	0.6	98	Small/Absent	Large (253.6)	253.6	Flighted
Egretta thula	Fa	C	Small (0.7)	0.7	97.3	Small/Absent	Large (289.3)	289.3	Flighted
Botaurus lentiginosus	Fa	C	Small (0.6)	0.7	113.9	Small/Absent	Large (248.0)	248	Flighted
Ixobrychus exilis	Fa	C	Absent (0.0)	0	68.5	Small/Absent	Medium (61.5)	61.5	Flighted
Pelecanus occidentalis	Fa	C	Medium (2.0)	4.7	239.6	Small/Absent	XL (1947.7)	1947.7	Flighted
Eudocimus ruber	Fa	AI	Small (0.5)	0.5	103	Small/Absent	Large (611.9)	611.9	Flighted
Ajaia ajaja	Fa	C	Small (0.5)	0.8	161.6	Small/Absent	XL (1543.0)	1543	Flighted
Mycteria americana	Fa	C	Small (0.2)	0.6	295.5	Small/Absent	XL (1800.0)	1800	Flighted
Anhinga anhinga	Fa	C	Small (0.1)	0.1	115.8	Small/Absent	Large (605.6)	605.6	Flighted
Phalacrocorax auritus	Fa	C	Small (0.9)	1.1	127.7	Small/Absent	Large (803.3)	803.3	Flighted
Morus bassanus	Fa	C	Small (0.9)	1.1	122.8	Small/Absent	XL (1731.3)	1731.3	Flighted
Calonectris diomedea	Fa	C	Medium (1.9)	1	53.1	Small/Absent	Large (404.9)	404.9	Flighted
Puffinus lherminieri	Fa	C	Small (0.8)	0.3	40.2	Small/Absent	Large (107.0)	107	Flighted
Puffinus gravis	Fa	C	Small (0.8)	0.6	72.4	Small/Absent	Large (380.0)	380	Flighted
Pterodroma hasitata	Fa	C	Small (0.7)	0.5	69	Small/Absent	Large (446.0)	446	Flighted
Oceanites oceanicus	Fa	C	Small (0.9)	0.4	42.8	Small/Absent	Large (144.8)	144.8	Flighted

Phoebastria immutabilis	Fa	C	Small (0.8)	1.5	180	Small/Absent	XL (2460.0)	2460	Flighted
Gavia immer	Fa	C	Medium (3.4)	5.3	157	Small/Absent	XL (1797.6)	1797.6	Flighted
Porzana carolina	Non-Fa	H	Medium (8.9)	3.4	38.2	Large	Medium (51.2)	51.2	Flighted
Fulica americana	Non-Fa	O	Large (16.5)	15.3	92.8	Large	Large (383.8)	383.8	Limited Flight
Gallinula chloropus	Non-Fa	H	Medium (8.7)	11	126.7	Large	Large (225.3)	225.3	Flighted
Laterallus jamaicensis	Fa	I	Medium (6.1)	1.2	19.7	Small/Absent	Medium (18.5)	18.5	Limited Flight
Rallus limicola	Fa	AI	Large (12.2)	5	40.9	Large	Large (129.7)	129.7	Limited Flight
Porphyrio martinica	Non-Fa	O	Medium (7.3)	3.5	47.7	Large	Large (163.9)	163.9	Limited Flight
Aramus guarauna	Fa	AI	Medium (8.3)	5.9	71	Large	Large (591.6)	591.6	Flighted
Phalaropus tricolor	Fa	AI	Large (11.5)	3.9	34	Large	Medium (61.2)	61.2	Flighted
Calidris canutus	Fa	I	Large (10.1)	4.1	40.6	Large	Large (102.6)	102.6	Flighted
Calidris melanotos	Fa	I	Medium (7.5)	1.9	25.5	Large	Medium (27.6)	27.6	Flighted
Calidris alba	Fa	I	Large (10.7)	3	28.1	Large	Medium (51.5)	51.5	Flighted
Calidris pusilla	Fa	I	Medium (9.5)	2.5	26.3	Large	Medium (22.5)	22.5	Flighted
Arenaria interpres	Fa	I	Medium (9.7)	4.1	42.5	Large	Medium (79.4)	79.4	Flighted
Gallinago undulata	Fa	I	Medium (8.5)	2.7	31.9	Large	Medium (69.3)	69.3	Flighted
Fratercula cirrhata	Fa	C	Small (0.7)	0.6	84.5	Large	Large (913.5)	913.5	Flighted
Sterna maxima	Fa	C	Medium (1.4)	0.9	65.8	Large	Large (260.5)	260.5	Flighted
Sterna hirundo	Fa	C	Medium (1.7)	0.6	36.4	Small/Absent	Medium (76.3)	76.3	Flighted
Sterna caspia	Fa	C	Small (1.0)	0.8	83.7	Large	Large (423.2)	423.2	Flighted
Sterna fuscata	Fa	C	Small (0.4)	0.2	46.9	Large	Large (195.0)	195	Flighted
Sterna antillarum	Fa	C	Small (0.9)	0.2	23	Small/Absent	Medium (24.8)	24.8	Flighted
Larus atricilla	Fa	C	Small (0.9)	0.5	54.6	Large	Large (267.5)	267.5	Flighted
Larus argentatus	Non-Fa	O	Medium (1.1)	1.1	96.5	Large	Large (787.9)	787.9	Flighted
Larus hyperboreus	Non-Fa	O	Small (0.8)	1	123.5	Small/Absent	XL (1132.0)	1132	Flighted
Larus californicus	Fa	I	Small (0.5)	0.5	106.4	Large	Large (470.0)	470	Flighted
Larus delawarensis	Non-Fa	O	Medium (1.2)	0.9	74.1	Large	Large (359.7)	359.7	Flighted
Rynchops niger	Fa	C	Medium (2.0)	0.9	44.7	Large	Large (288.2)	288.2	Flighted

Charadrius vociferus	Fa	I	Large (11.4)	2.8	24.6	Large	Medium (25.6)	25.6	Flighted
Recurvirostra americana	Non-Fa	O	Medium (9.0)	6.7	74.3	Large	Large (320.5)	320.5	Flighted
Haematopus palliatus	Fa	AI	Medium (5.3)	4.9	93.2	Large	Large (461.0)	461	Flighted
Pluvialis dominica	Fa	I	Medium (2.8)	2.3	81.3	Large	Large (158.6)	158.6	Flighted
Columba livia	Non-Fa	Gr/Fr	Small (0.6)	0.5	84.3	Large	Large (286.0)	286	Flighted
Streptopelia capicola	Non-Fa	Gr/Fr	Small (0.4)	0.2	52	Large	Large (119.4)	119.4	Flighted
Zenaida asiatica	Non-Fa	Gr/Fr	Absent (0.0)	0	56	Large	Large (140.1)	140.1	Flighted
Zenaida macroura	Non-Fa	Gr/Fr	Absent (0.0)	0	49.5	Small/Absent	Large (100.7)	100.7	Flighted
Columbina passerina	Non-Fa	Gr/Fr	Absent (0.0)	0	31.3	Small/Absent	Medium (32.3)	32.3	Flighted
Opisthocomus hoazin	Non-Fa	H	Medium (4.9)	4	82.2	Large	Large (750.0)	750	Limited Flight
Numida meleagris	Non-Fa	H	Large (15.0)	39.4	262.7	Large	XL (1351.0)	1351	Flighted
Gallus gallus	Non-Fa	Gr/Fr	Medium (9.6)	18.3	191.5	Large	XL (2045.0)	2045	Flighted
Bonasa umbellus	Non-Fa	O	Large (40.1)	41.8	104.3	Large	Large (397.9)	397.9	Flighted
Canachites canadensis	Non-Fa	H	Large (34.5)	40	116	Large	Large (576.0)	576	Limited Flight
Tetrao urogallus	Non-Fa	Gr/Fr	Large (29.4)	52	177	Large	XL (2500.0)	2500	Limited Flight
Centrocercus urophasianus	Non-Fa	Gr/Fr	Large (49.2)	73	148	Large	XL (1427.0)	1427	Flighted
Dendragapus obscurus	Non-Fa	Gr/Fr	Large (36.2)	50	138	Large	XL (1250.0)	1250	Limited Flight
Tympanuchus phasianellus	Non-Fa	H	Large (36.2)	49	135.5	Large	Large (850.0)	850	Flighted
Tympanuchus cupido	Non-Fa	Gr/Fr	Large (34.4)	45	131	Large	XL (1120.0)	1120	Flighted
Lagopus lagopus	Non-Fa	H	Large (45.4)	47	103.5	Large	Large (525.0)	525	Limited Flight
Lagopus mutus	Non-Fa	H	Large (40.9)	47	115	Large	Large (502.0)	502	Flighted
Meleagris gallopavo	Non-Fa	O	Large (20.0)	46.5	232.8	Large	XL (6775.0)	6775	Flighted
Chrysolophus pictus	Non-Fa	Gr/Fr	Large (13.2)	9.6	72.7	Large	Large (366.7)	366.7	Limited Flight
Perdix perdix	Non-Fa	Gr/Fr	Large (28.2)	19	67.3	Large	Large (395.0)	395	Limited Flight
Alectoris graeca	Non-Fa	Gr/Fr	Large (22.1)	19	86	Large	Large (576.0)	576	Limited Flight
Coturnix japonica	Non-Fa	Gr/Fr	Large (16.6)	26.3	158.4	Large	Medium (90.0)	90	Flighted
Oreortyx pictus	Non-Fa	Gr/Fr	Large (19.9)	17.5	88	Large	Large (250.0)	250	Limited Flight

Callipepla squamata	Non-Fa	Gr/Fr	Large (15.3)	10.5	68.5	Large	Large (175.0)	175	Flighted
Callipepla californica	Non-Fa	Gr/Fr	Large (16.2)	13.9	86	Large	Large (173.0)	173	Flighted
Callipepla gambelii	Non-Fa	Gr/Fr	Large (12.6)	11.3	89	Large	Large (188.0)	188	Flighted
Colinus virginianus	Non-Fa	O	Large (15.1)	11.3	74.7	Large	Large (161.1)	161.1	Flighted
Cyrtonyx montezumae	Non-Fa	O	Large (21.5)	11.5	53.5	Large	Large (186.0)	186	Flighted
Cairina moschata	Non-Fa	O	Medium (9.3)	12.8	137.9	Large	Large (585.0)	585	Flighted
Aix sponsa	Fa	C	Medium (8.1)	8.9	109.5	Large	Large (537.3)	537.3	Flighted
Anas fulvigula	Fa	C	Medium (8.9)	12.6	142	Large	Large (543.9)	543.9	Flighted
Anas platyrhynchos	Non-Fa	Gr/Fr	Large (11.5)	16.1	169.5	Large	Large (929.5)	929.5	Flighted
Anas americana	Non-Fa	H	Large (15.6)	22.5	144.4	Large	Large (530.0)	530	Flighted
Anas discors	Non-Fa	Gr/Fr	Medium (6.2)	6.6	106.1	Large	Large (250.3)	250.3	Flighted
Aythya collaris	Non-Fa	H	Large (10.5)	13.3	126.9	Large	Large (612.1)	612.1	Flighted
Mergus serrator	Fa	C	Medium (1.9)	2.8	144.6	Large	Large (461.4)	461.4	Flighted
Anser cygnoides	Non-Fa	H	Large (11.9)	90.2	758.0	Large	XL (3150.0)	3150	Flighted
Struthio camelus	Non-Fa	H	Medium (6.5)	94	1440	Large	XL (122000)	122000	Flightless
Dromaius novaehollandiae	Non-Fa	O	Medium (2.7)	8.6	312	Large	XL (38300)	38300	Flightless
Apteryx mantelli	Fa	I	Large (12.2)	16.5	135.1	Large	XL (2012)	2012	Flightless
Rhea americana	Non-Fa	H	Large (26.7)	48	180	Large	XL (8500)	8500	Flightless
Crypturellus tataupa	Non-Fa	Gr/Fr	Medium (3.0)	1.25	42.3	Large	Large (220)	220	Limited Flight
Nothoprocta cinerascens	Fa	I	Medium (4.7)	2.4	51.3	Large	Large (526)	526	Limited Flight
Nothoprocta ornata	Non-Fa	Gr/Fr	Medium (2.3)	18.7	82.4	Large	Large (514)	514	Limited Flight
Nothura darwini	Non-Fa	Gr/Fr	Medium (2.3)	14.1	60.8	Large	Large (234)	234	Limited Flight
Nothura maculosa	Non-Fa	Gr/Fr	Medium (6.5)	3.1	48	Large	Large (247)	247	Limited Flight
